# Pharmacokinetic evaluation and in vivo anti-inflammatory potential of cyanoacetate derivatives of succinimide

**DOI:** 10.1007/s00210-026-05720-z

**Published:** 2026-07-22

**Authors:** Aini Pervaiz, Muhammad Saeed Jan, Ali Khan, Bushra Ansari, Imad Ahmad, Wadood Ali Shah, Syed Muhammad Mukarram Shah, Abdur Rauf, Abdullah S. M. Aljohani, Waleed Al Abdulmonem, Marcello Iriti

**Affiliations:** 1 The Professional Institute of Health Sciences, Mardan, Khyber Pakhtunkhwa Pakistan; 2 Department of Pharmacy, Bacha Khan University, Charsadda, 24420 Khyber Pakhtunkhwa Pakistan; 3 Department of Pharmacy, University of Swabi, Swabi, Khyber Pakhtunkhwa Pakistan; 4 Department of Pharmacy, University of Malakand, Chakdara, Khyber Pakhtunkhwa Pakistan; 5 Department of Chemistry, University of Swabi, Swabi, 23430 Anbar, Khyber Pakhtunkhwa Pakistan; 6 Department of Medical Biosciences, College of Veterinary Medicine, Qassim University, Buraydah, Saudi Arabia; 7 Department of Pathology, College of Medicine, Qassim University, Buraydah, Saudi Arabia; 8 Department of Biomedical, Surgical and Dental Sciences, University of Milan, Via Luigi Vanvitelli 32, 20129 Milan, Italy; 9 National Interuniversity Consortium of Materials Science and Technology (INSTM), 50121 Florence, Italy

**Keywords:** Anti-inflammatory, Succinamide, Prostaglandin, Histamine, Bradykinin, ADME studies

## Abstract

The purpose of the study is to evalute the in vivo anti -inflammatory activity of newly synthesized cyanoacetate derivatives of succinimides (C-23, C-31, and C-44) and to elucidate their possible mechanisms of action through key inflammatory mediator pathways. Anti-inflammatory activity was assessed using carrageenan-induced paw oedema and arachidonic acid-induced ear oedema models in vivo. Mechanistic involvement was investigated through histamine-, prostaglandin-, leukotriene-, and bradykinin-induced paw oedema assays. In silico ADME studies were performed to predict pharmacokinetic properties and metabolic interactions. All tested compounds exhibited significant anti-inflammatory activity, with C-44 showing the highest potency. Mechanistic studies indicated that the anti-inflammatory effects were predominantly mediated via inhibition of prostaglandin and leukotriene pathways. ADME predictions suggested high gastrointestinal absorption, favourable oral bioavailability, and minimal interaction with major metabolic enzymes. The synthesized cyanoacetate derivatives, particularly C-44, demonstrate promising anti-inflammatory potential with favourable pharmacokinetic profiles, supporting their candidacy for further development as effective anti-inflammatory agents.

**Supplementary Information:**

The online version contains supplementary material available at https://doi.org/10.1007/s00210-026-05720-z.

## Introduction

Inflammation is a pivotal response orchestrated by the immune system against infection and tissue damage, with implications spanning from arthritis to cancer, stroke pathogenesis, and neurological and cardiovascular diseases (Ricciotti and FitzGerald 2011). Its multifaceted role encompasses tissue regeneration, immunological surveillance, and healing following injury (Vodovotz et al. 2008). It is emphasize that the inflammatory process protects our bodies from disease by releasing mediators and cells that respond to foreign chemicals and anticipate them. While often associated with infection, inflammation does not universally accompany all infections (Panes et al. 2013). Manifesting through hallmark signs of warmth, redness, swelling, and pain, inflammation entails a cascade involving vasodilation, the influx of chemical mediators, phagocytic cells, and enhanced vascular permeability (Punchard et al. 2004).

Acute inflammation, lasting from minutes to days depending on severity, acts as an innate immune response to eliminate injurious agents and initiate tissue repair (Sutton et al. 2013) and (Karin and Clevers 2016). In contrast, chronic inflammation, typified by prolonged mononuclear cell infiltration and impaired healing, is distinctively linked to tissue dysfunction rather than acute triggers like injury or infection (Zigmond et al. 2012) and (Medzhitov 2008). The development of anti-inflammatory medications, notably nonsteroidal anti-inflammatory drugs (NSAIDs), has historically targeted the cyclooxygenase (COX) enzymes, with aspirin’s discovery as a COX inhibitor marking a pivotal milestone (Vane 1971; Munir et al. 2022). Subsequent advancements identified COX-2 as a separate isozyme, prompting the search for safe drugs specifically inhibiting its activity (Munir et al. 2022). Mechanistically, these drugs modulate arachidonic acid metabolism, impacting mediators including bradykinin, histamine, prostaglandins, and leukotrienes (Al-Mulla 2017).

The hererocyclic scaffolds have attracted increasing attention as the modulators effect of inflamamotry pathways, particulary due to their structure versitlity and favourable pharmacological profiles. The succinimides and their derivatives containing nitrogenous ring having a well-known pharmacological history. Literature survey revealed that various aldehyde (Sadiq and Nugent 2020), ketone (Jan et al. 2020), ketoesters (Sadiq et al. 2015; Alqahtani et al. 2022), and *N*-aryl analogues of succinimides are reported for various chemical and biological purposes. Our previous studies demonstrated that selected succinimide analogue effectivein in vitro inhibitory activity against COX-1, COX-2, and 5-LOX key enzyme implicated in inflammatory mediators biosynthesis (Pervaiz et al. 2022). The succinimide derivatives are also explored for anti-diabetic potential which were related to inflammation as well (Mahnashi et al. 2023; Talib et al. 2023). The succinimide derivatives were also exhibited antioxidant, anticholinesterases, anti-α-amylase, and anti-α-glucosidase potential (Alshehri et al. 2022). Based of the aforementioned multitargeted approaches, the present study delves into the in vivo anti-inflammatory potential of cyanoacetate derivatives of succinimide, an area gaining traction due to its promising role in inflammation modulation (Liu et al. 2022). These three cyano-acetate derivatives of succinimide including compound 23 Methyl 2-cyano-2-(2,5-dioxopyrrolidin-3-yl)acetate, compound 31 Methyl 2-cyano-2-(1-methyl-2,5-dioxopyrrolidin-3-yl)acetate, and compound 44 Methyl 2-cyano-2-(1-ethyl-2,5-dioxopyrrolidin-3-yl) exhibited anti-inflammatory via inhibiting COX-2 (IC50 value of 204.08, 68.60, and 50.93 mM, respectively); COX-1 (IC50 value of 287, 185, and 143 mM, respectively); and 5-LOX (IC50 value of 138, 50.76, and 20, 87 mM respectively). They exhibited choline-mimetic potential, such as compound 23, 31, and 44 inhibited AChE enzyme (IC50 value of 240, 174, and 134 mM, respectively) and BChE enzyme (IC50 value of 203, 134, and 97 mM, respectively). The compounds 23, 31, and 44 exhibited anti-diabetic effect via inhibiting a-amylase enzyme (IC50 values of 250, 106, and 60 mM, respectively). Molecular docking studies revealed that the synthesized compounds have good binding affinity in the binding pockets of AChE, BChE, COX-2, 5-LOX, and a-amylase enzyme and showed high binding energies. Compound 44 and 31 showed strong anti-inflammatory and anti-diabetic activity while they displayed moderate anti-cholinesterase activity supported by molecular docking results (Pervaiz et al. 2022). The incorporation of a cyano substituent is hypothesized to enhance pharmacokinetic properties, metabolic stability, and enzyme-binding affinity, thereby strengthening anti-inflammatory efficacy. This study aims to evaluate these compounds’ efficacy in attenuating inflammation in vivo and elucidate their underlying mechanisms of action, thereby contributing to the development of targeted anti-inflammatory therapies.

## Materials and methods

### Chemical and drugs

For the in vivo inflammatory study, we meticulously sourced arachidonic acid (CAS No. 506–32-1) and carrageenan (CAS No. 9064–67-7) from Sigma Aldrich Germany and Cayman Chemical USA, respectively, from Labcare Enterprises Peshawar, KP, Pakistan. Our choice of positive controls, nimesulide, diclofenac, and aspirin, exemplifies our commitment to rigour and reliability, procured from industry leaders Hilton Pharmaceuticals and Zafa Pharmaceuticals Hayatabad Peshawar, KP, Pakistan.

We acquired a suite of phlogistic agents from reputable suppliers to elucidate the mechanistic insights of our synthesized compounds. Bradykinin (EEC No. 227–781-2), histamine (CAS No. 51–45-6), leukotriene (CAS No. 71160–24-2), and prostaglandin (CAS No. 14010) were meticulously sourced from Sigma–Aldrich Germany. Cetirizine, a pivotal standard drug in histamine-induced inflammation, was secured from Alliance Pharmaceuticals (Pvt.) Limited, Peshawar, KP, Pakistan. We sourced Icatibant (CAS No. 138614–30-9), a potent bradykinin antagonist, from Sigma–Aldrich for targeted antagonistic studies. Moreover, our study benefited from the inclusion of Montelukast, provided by Pfizer Pharmaceuticals (Pvt.) Limited, Karachi, Pakistan, serving as a pivotal positive control in the leukotriene inflammatory assay.

### Experimental animals

This study employed Albino mice of either sex weighing 25–30 g, sourced from the National Institute of Health (NIH), Islamabad. Meticulous care was taken to ensure their well-being, with the mice housed in hygienic cages under standard laboratory conditions. These conditions included a controlled humidity of 50% ± 5%, temperature maintained at 25 ± 2 °C, and a light–dark cycle comprising 12 h of light followed by 12 h of darkness. The mice were provided with standard mouse feed and tap water ad libitum access.

All in vivo experiments were conducted under stringent human oversight, adhering to the highest ethical standards. Before commencement, the procedures and protocols were subjected to rigorous review and approval by the Institutional Research Ethical Committee of the Department of Pharmacy, University of Swabi, Pakistan, via DREC/02–2020/005, ensuring compliance with ethical guidelines and animal welfare regulations (Zeb et al. 2016). Throughout the study, the animals were treated with utmost care and respect, with each mouse being used only once before being euthanized following established ethical practices. Furthermore, with respect to biological variability, animals were randomly assigned to treatment groups with sex balanced across groups to minimize sex-related bias.

### Acute toxicity

To assess acute toxicity, the animals of either sex were divided into different groups, including control and test groups, each consist of mice (*n* = 8). The synthesized compounds were orally administered at varying doses ranging from 5 to 2000 mg/kg body weight. Following administration, all animals were closely monitored for 72 h for any signs of adverse effects such as spontaneous activity, allergic symptoms, cyanosis, catalepsy, convulsions, aggressiveness, writhing, tail pinch response, ataxia, and abnormal behavior, following the protocol outlined by (Lorke 1983).

### Carrageenan induced inflammation

The anti-inflammatory efficacy of the synthesized compounds was evaluated in Swiss albino mice of both sexes, weighing from 25 to 30 g, as per the protocol outlined by Subhan et al. (2007). Forty mice were randomly allocated to one of five groups, each having mice *n* = 8. Group I served as the negative control and received a solution containing 10% DMSO, normal saline, and Tween-80 in a ratio of 5:94:1 (v/v) used for preparation of the dosing solution. Specifically, the vehicle consisted of 10% DMSO, 1% Tween-80, and 94% normal saline (v/v), resulting in a final vehicle volume of 100% administered at a dosage of 10 ml/kg body weight. Diclofenac sodium, administered at 10 mg/kg, served as the positive control in Group II. The synthesized compounds were administered intraperitoneally to Groups III, IV, and V at 10, 20, and 30 mg/kg body weight, respectively.

To induce inflammation, a solution of 2% carrageenan in saline (0.2 ml) was administered intraplantarly in hind paw to all groups after 30 min, following the protocol by Alqahtani et al. (2022). The degree of inflammation was assessed using a plethysmometer (Model: LE 7500 plan lab S.L) before introducing carrageenan and then re-measured after carrageenan injection at time intervals of 1, 2, 3, 4, and 5 h.

The percentage inhibition of paw oedema was calculated using the following formula:

$$Percent\;inhibition\;\left(\%\right)=\;Cg-Tg/Cg\times100$$

where:

Tg = paw volume of test group.

Cg = control group inflammation.

### Arachidonic acid induced inflammation assay

The ear oedema test, as delineated by Romay et al. (2000), formed the cornerstone of our investigation. Mice were subjected to a 20-h fasting period with unrestricted access to distilled water. Random assignment placed 8 mice of either sex into five distinct groups.

Arachidonic acid-induced ear oedema was initiated by the topical application of 2 mg/kg arachidonic acid dissolved in acetone onto the ear of each mouse, while acetone alone served as the control, applied to the contralateral ear. Preceding arachidonic acid administration, the synthesized compounds’ varying concentrations (10, 20, and 30 mg/kg body weight) were topically applied.

Nimesulide (100 mg/kg) served as the positive control. Following arachidonic acid induction, ear thickness measurements were taken using a vernier caliper at 10, 15, 30, 45, and 60 min. Subsequently, the animals were euthanized, and bilateral ear samples were collected and weighed to determine percent inhibition relative to the control, as detailed by Jaijoy et al. (2010).

$$\%\;Inhibition\;=\frac{\left[\left(Wc-Wt\right)\right]}{Wc}\times100$$

where Wc represents the mean ear weight of the control group and Wt represents the mean ear weight of the treated group.

### Possible anti-inflammatory mechanism

To unravel the potential anti-inflammatory mechanism of the newly synthesized compounds, we investigated their effects on various inflammatory mediators, including prostaglandin, histamine, bradykinin, and leukotriene.

Prostaglandins play a crucial role in the inflammatory process by mediating pain and fever. Histamine, released during allergic reactions, contributes to vasodilation and increases vascular permeability. Bradykinin, a potent vasodilator, induces pain and inflammation. Leukotrienes, derived from arachidonic acid, are powerful mediators of inflammation, contributing to bronchoconstriction and increasing vascular permeability.

By examining the compounds’ influence on these critical inflammatory mediators, we aimed to elucidate their mode of action and potential therapeutic relevance in mitigating inflammation. This comprehensive approach sheds light on the intricate interplay between the synthesized compounds and the inflammatory cascade, paving the way for developing novel anti-inflammatory agents with enhanced efficacy and specificity.

### Compounds’ effects on histamine-induced inflammation

To assess the effects of the synthesized compounds on histamine-induced inflammation, mice were stratified into four groups, comprising both sexes, with each group consisting of 8 mice; histamine was given intraplantarly. Group I received 0.1 mL of 1% histamine solution prepared in normal saline (1 mg/ml). Group II received chlorpheniramine maleate at a dose of 10 mg/kg body weight and histamine administration. Group III received histamine and the tested compounds (C-23, C-31, and C-44) at a 30 mg/kg body weight each. Group IV served as the control and received 0.9% normal saline. Following histamine administration, paw oedema volume was evaluated at 1, 2, 3, 4, and 5 h post-induction (Ndebia et al. 2011). This systematic approach allowed us to assess the potential anti-inflammatory effects of the synthesized compounds in the context of histamine-induced inflammation, providing valuable insights into their therapeutic utility.

### Bradykinin-induced inflammation

In our investigation of bradykinin-induced inflammation when administered intraplantarly, mice were allocated into four distinct groups, each containing 8 animals. Group I received 10% DMSO, while Group II was administered 20 µg/mL of bradykinin. Group III received bradykinin and icatibant at a 1 mg/kg dose. Group IV received the synthesized compounds C-23, C-31, and C-44 at a dose of 30 mg/kg body weight each, alongside bradykinin.

Following bradykinin administration, paw oedema volume was assessed for all animals. This systematic approach allowed us to evaluate the potential anti-inflammatory effects of the synthesized compounds in the context of bradykinin-induced inflammation, providing valuable insights into their therapeutic efficacy (Marceau et al. 1998).

### Prostaglandin-induced inflammation

In our investigation of the effects of intraplantarly administered prostaglandin-induced inflammation, mice were stratified into four distinct groups. Group I served as the negative control and received 0.9% NaCl. Group II was administered 0.01 µg/mL of prostaglandin E_2_ (PGE_2_). Group III received celecoxib and prostaglandin at a dose of 50 mg/kg body weight. Group IV was administered the synthesized compounds C-23, C-31, and C-44 at 30 mg/kg body weight each, alongside prostaglandin. Following prostaglandin administration, paw oedema volume was measured at 1–5 h post-injection for all mice. This rigorous methodology allowed us to assess the potential anti-inflammatory effects of the synthesized compounds in the context of prostaglandin-induced inflammation, providing valuable insights into their therapeutic potential (Mukarram Shah 2015).

### Leukotriene-induced inflammation

In our investigation of intraplantarly administerd leukotriene-induced inflammation, mice were randomly assigned to four distinct groups. Group I served as the control and received 0.9% normal saline. Group II was administered 10 µg/mL of leukotriene. Group III received the standard drug, montelukast, at a dose of 20 mg/kg body weight, along with leukotriene. Group IV was administered the synthesized compounds C-23, C-31, and C-44 at 30 mg/kg body weight each, alongside leukotriene.Following leukotriene administration, paw oedema volume was measured 1–5 h post-treatment using a plethysmometer. The paw oedema volume of both control and treated groups was compared with that of the standard group of animals, allowing for a comprehensive evaluation of the anti-inflammatory effects of the synthesized compounds in the context of leukotriene-induced inflammation (Rao et al. 2007).

### Statistical analysis

Each experiment was repeated three times, and the results were expressed as mean ± standard error of the mean (SEM). Prior to inferential analysis, data were assessed for normality and homogeneity of variance using appropriate diagnostic tests to ensure compliance with the assumptions of parametric analysis. Statistical analysis was performed using two-way analysis of variance (ANOVA) followed by Bonferroni correction to adjust for multiple pairwise comparisons within each experimental model, and all statistical analyses were performed independently for each assay. Dose-dependent effects for the tested doses (10, 20, and 30 mg/kg) were evaluated by analyzing the main effect of dose using two-way ANOVA, followed by Bonferroni post hoc comparisons of each dose against the vehicle control at corresponding time points. All statistical analyses were carried out using GraphPad Prism software (version 3, GraphPad Software, San Diego, CA, USA). Effect sizes (η^2^) were calculated for ANOVA outcomes where applicable to estimate the magnitude of treatment effects. A *P* value less than 0.05 was considered statistically significant.

### Pharmacokinetic evaluation

Drug-like properties encompass various physicochemical characteristics, such as water solubility and lipophilicity, along with assessments of drug similarity, pharmacokinetic profile, and medicinal chemistry. In evaluating succinimide derivatives, these properties were rigorously examined by Ahmad et al. (2023) and Daina et al. (2017). For the analysis of 2D structures and the implementation of string-based searches, the SwissADMET database serves as a valuable resource. Researchers can use this database to input search strings for various ligands, enabling efficient retrieval and analysis of pertinent information.

## Results

Our research study includes the following three compounds. On these three compounds, different activities were performed.

The synthesized succinimide derivatives used in this study are summarized in Table 1, which lists the compound codes, IUPAC names, chemical structures, and SMILES notations. These details provide a clear reference for the chemical identity of each ligand and ensure reproducibility in subsequent computational and biological evaluations. All SMILES notations were verified against the corresponding structures to confirm their accuracy for molecular docking and in silico analyses.

**Table 1:**
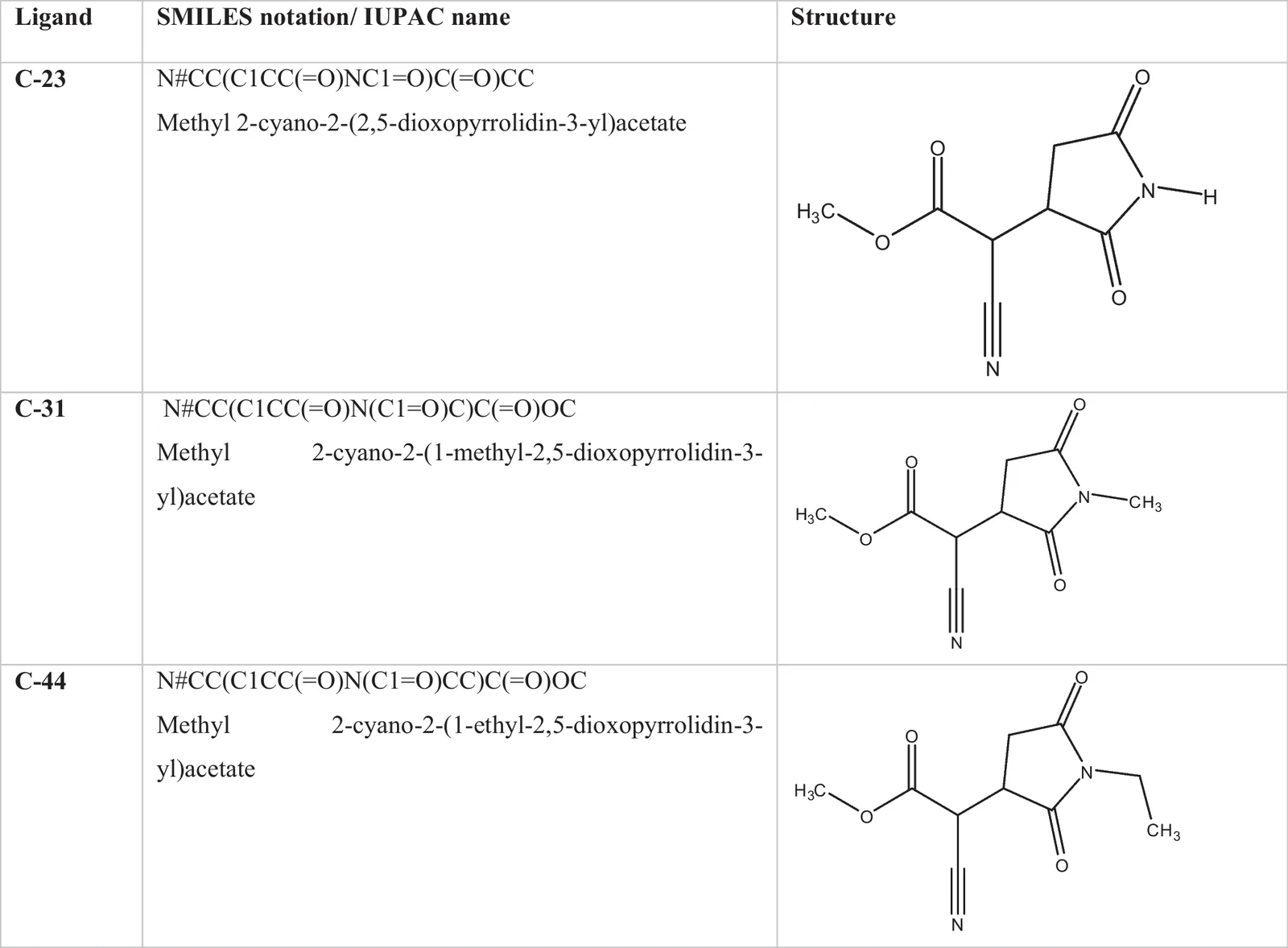
Compound codes/ligands, IUPAC name, chemical structure, and SMILES notation of succinimide derivatives

### General synthesis

**Fig. 1:**
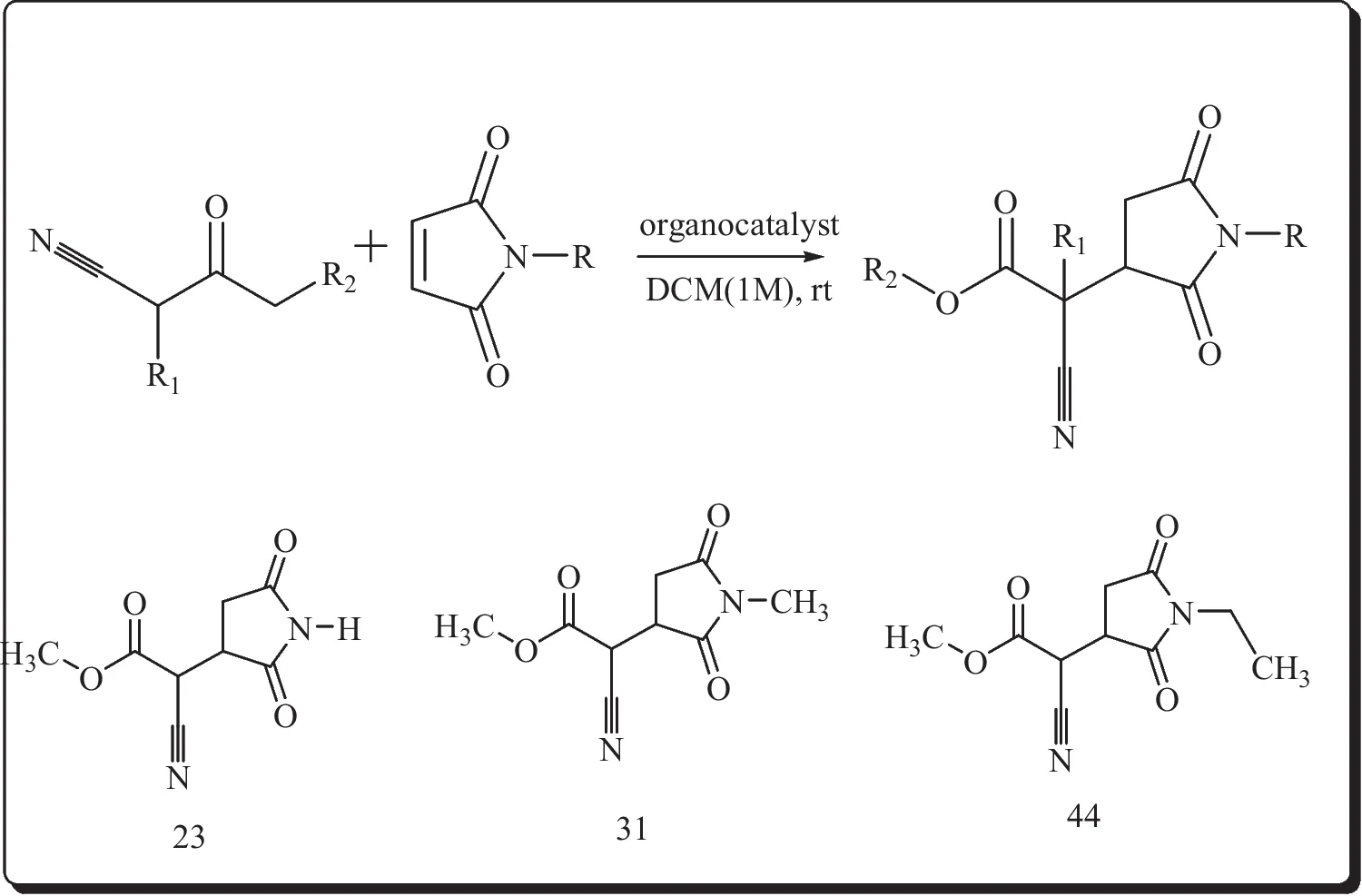
Common method for synthesis of compounds and compound structures

In our study, various cyanoacetate was added to different functionalities of maleimides in the presence of an organocatalyst (Fig. 1). The reaction progress was tracked using thin-layer chromatography. Water was applied to quench the reaction after it had completed conversion. The water layer was isolated from the organic layer using a separating funnel. The organic evaporated to dryness using a rotary evaporator under low vacuum. Column chromatography containing eluents including ethyl acetate and n-hexane was used to purify the crude reaction mixture. The chosen solvent fraction was used in column chromatography to isolate pure compound(s). ^1^H NMR and ^13^CNMR were used to validate the structures of pure isolated compounds.

### Acute toxicity

In acute toxicity research, no mortalities were observed in mice administered at the oral dose up to 1000 mg/kg body weight for any of the tested compounds (Table 1). Throughout the 24-h observation period, animals were monitored for behavioral, neurological, and physiological signs of toxicity, including writhing, ataxia, grooming, and locomotor activity; no significant adverse effects were observed, indicating good tolerability. Accordingly, the median lethal dose (LD_50_) of all compounds is reported as > 1000 mg/kg. According to the OECD guidelines for acute oral toxicity, an LD_50_ dose greater than 300 mg/kg and up to 2000 mg/kg categorizes the substance as category IV, indicating a low level of toxicity as shown in Table 2 (Gribaldo et al. 2005). Based on these findings, all tested compounds were considered safe for subsequent in vivo pharmacological evaluation (Table 2).

**Table 2:** Acute toxicity results

Group	No of mice	Compounds dose (mg/kg)
1	8	5
2	8	25
3	8	50
4	8	100
5	8	200
6	8	300
7	8	400
8	8	500
9	8	1000
10	8	2000

### In vivo anti-inflammatory assays

#### Carrageenan-induced inflammatory activity

All tested compounds produced a marked reduction in carrageenan-induced paw oedema compared with the vehicle control. Three dose levels (10, 20, and 30 mg/kg) were evaluated for each compound (Table 3). While all compounds demonstrated dose-dependent anti-inflammatory effects, compound C-44 consistently showed the strongest inhibition across time points, particularly at 30 mg/kg. To improve readability, repetitive descriptions of C-44’s superiority were minimized, and comparative performance across all tested compounds is emphasized. The proposed mechanism of action for compound C-44 might involve inhibiting pro-inflammatory cytokines and suppressing the NF-κB signaling pathway, which plays a crucial role in the inflammatory response (Kim et al. 2013).

**Table 3:** Anti-inflammatory potential of the newly synthesized compounds in carrageenan-induced paw oedema volume

Test compounds and dose (mg/kg)	% Inhibition of paw oedema				
	1st h	2nd h	3rd h	4th h	5th h
Vehicle	7.22 ± 0.19	7.61 ± 0.46	7.46 ± 0.34	7.88 ± 0.35	7.82 ± 0.37
Diclofenac (10 mg/kg)	47.2 ± 0.80	54.6 ± 1.10	55.4 ± 0.75	56.26 ± 1.36	57.3 ± 1.25
C-23 (10 mg/kg)	31.1 ± 0.79^***^	33.36 ± 0.97^***^	19.66 ± 0.68^***^	31.4 ± 0.72^***^	31.2 ± 0.82^***^
C-23 (20 mg/kg)	34.5 ± 0.72^***^	34.56 ± 0.76^***^	21.8 ± 0.62^***^	29.56 ± 0.61^***^	31.83 ± 0.31^***^
C-23 (30 mg/kg)	38.76 ± 0.83^**^	39.2 ± 0.85^**^	34.4 ± 0.62^**^	37.6 ± 0.80^**^	40.56 ± 0.84^**^
C-31 (10 mg/kg)	42.9 ± 0.66^*^	44.5 ± 0.92^*^	43.5 ± 0.94^*^	39.4 ± 0.83^**^	39.6 ± 0.91^**^
C-31 (20 mg/kg)	44.6 ± 0.79^ns^	46.5 ± 0.60^ns^	42.6 ± 0.58^*^	34.6 ± 0.60^**^	41.3 ± 0.56_**_
C-31 (30 mg/kg)	47.3 ± 0.25^ns^	51.6 ± 0.61^ns^	55.0 ± 0.90^ns^	49.2 ± 0.99^ns^	48.42 ± 0.88^ns^
C-44 (10 mg/kg)	44.3 ± 0.7^*^	45.2 ± 0.43^*^	41.16 ± 0.90^**^	39.86 ± 0.41^**^	40.22 ± 0.36^**^
C-44 (20 mg/kg)	45.6 ± 0.95^ns^	50.4 ± 0.85^ns^	50.3 ± 0.85^ns^	53.67 ± 0.87^ns^	48.63 ± 0.80^ns^
C-44 (30 mg/kg)	46.4 ± 0.81^ns^	54.2 ± 0.72^ns^	52.67 ± 0.86^ns^	55.6 ± 0.60^ns^	56.5 ± 0.55^ns^

Values are expressed as mean ± SEM, (*n* = 6). Data analysis was conducted using one-way ANOVA followed by Bonferroni post hoc test comparing each compound directly to diclofenac. Significance levels vs diclofenac: *ns* not significant, *P < 0.05, **P < 0.01, ***P < 0.001. The statistical significance levels (using Graph Pad Prism 5) were as followed

#### Inflammation induced by arachidonic acid (AA)

The investigated compounds demonstrated marked efficacy in reducing AA-induced topical inflammation at 10, 20, and 30 mg/kg doses (Table 4). Notably, compound C-44 emerged as the most potent among all tested compounds. The C-44 substantially reduced paw oedema volume at various doses. The anti-inflammatory activity of C-44 closely approximates that of the standard drug used in this assay.

**Table 4:** Potential of the tested compounds for anti-inflammatory activity in ear oedema induced by arachidonic acid

Test compounds and dose (mg/kg)	% Inhibition of ear oedema				
	10 min	15 min	30 min	45 min	60 min
Negative control	7.22 ± 0.19	7.61 ± 0.46	7.46 ± 0.34	7.88 ± 0.35	7.82 ± 0.37
Nimesulide	58.4 ± 0.65	61.27 ± 0.47	63.57 ± 0.45	66.47 ± 0.55	67.63 ± 0.50
C-23 (10 mg/kg)	34.63 ± 0.57^***^	35.53 ± 0.60^***^	38.5 ± 0.56^***^	29.26 ± 0.70^***^	27.46 ± 0.55^***^
C-23 (20 mg/kg)	40.63 ± 0.57^***^	39.5 ± 0.556^***^	40.73 ± 0.58^***^	31.62 ± 0.72^***^	30.46 ± 0.75^***^
C-23 (30 mg/kg)	43.73 ± 0.58^***^	46.33 ± 0.71^***^	49.4 ± 0.73^***^	37.83 ± 0.61^***^	30.36 ± 0.90^***^
C-31 (10 mg/kg)	38.73 ± 0.58^**^	42.46 ± 0.60^**^	46.63 ± 0.65^**^	47.66 ± 0.55^**^	42.33 ± 0.75^**^
C-31 (20 mg/kg)	48.3 ± 0.665^*^	57.3 ± 0.6^ns^	59.23 ± 0.61^ns^	55.56 ± 0.70^ns^	53.65 ± 0.73^ns^
C-31 (30 mg/kg)	52.66 ± 0.75^ns^	58.4 ± 0.43^ns^	60.63 ± 0.61^ns^	59.60 ± 0.72^ns^	56.56 ± 0.77^ns^
C-44 (10 mg/kg)	48.6 ± 0.56^*^	52.16 ± 0.70^*^	54.4 ± 0.65^ns^	54.46 ± 0.75^ns^	56.7 ± 0.76^ns^
C-44 (20 mg/kg)	52.6 ± 0.89^ns^	55.46 ± 0.70^ns^	58.66 ± 0.72^ns^	62.66 ± 0.61^ns^	62.36 ± 0.60^ns^
C-44 (30 mg/kg)	56.56 ± 0.60^*^	36.47 ± 0.40^***^	61.42 ± 0.56^ns^	59.51 ± 0.63^ns^	64.66 ± 0.58^ns^

Values are expressed as mean ± SEM, (*n* = 6). Data analysis was conducted using one-way ANOVA followed by Bonferroni post hoc test comparing each compound directly to nimesulide. Significance levels vs diclofenac: *ns* not significant, *P < 0.05, **P < 0.01, ***P < 0.001. The statistical significance levels (using Graph Pad Prism 5) were as followed

The proposed mechanism of action for C-44 involves the inhibition of pro-inflammatory mediators such as TNF-α and IL-6, which are critical in the inflammatory cascade (Veber et al. 2002). This inhibition likely results from C-44’s interaction with key signalling pathways, reducing the expression of these cytokines and thereby mitigating inflammation. The efficacy of C-44 in this model suggests its potential as a novel anti-inflammatory agent with comparable effectiveness to established treatments.

#### Anti-inflammatory mechanism of compounds

We conducted a comprehensive analysis to elucidate the potential anti-inflammatory mechanisms of the evaluated substances, focusing on potent and dose-dependent proinflammatory agents such as bradykinin, histamine, prostaglandin, and leukotriene. These mechanistic insights are based on pharmacological responses observed in established in vivo inflammation models and should be interpreted as inferred mechanisms rather than experimentally validated molecular targets.

#### Involvement of histamine as an inflammatory mediator

Histamine was examined explicitly as a critical inflammatory mediator to assess the anti-inflammatory activity of compounds C-23, C-31, and C-44 at a dose of 30 mg/kg. Using a histamine-induced inflammatory response model, these compounds demonstrated potent anti-inflammatory activity (Table 5). Freshly prepared 0.1 ml of 1% histamine (1 mg/ml) in normal saline was given to mice. Among them, C-44 showed superior efficacy compared to the other compounds studied. The results indicated that the tested compounds exhibited moderate anti-histaminic activity, highlighting their potential as effective anti-inflammatory agents.

**Table 5:** Compounds with anti-inflammatory potency against histamine-induced inflammation

Treatment/dose (mg/kg)	% Inhibition of paw oedema				
	1st h	2nd h	3rd h	4th h	5th h
Negative control	2.73 ± 0.04	2.41 ± 0.07	2.24 ± 0.03	1.96 ± 0.04	2.20 ± 0.07
CPM (25 mg/kg)	72.36 ± 0.56	55.53 ± 0.50	63.36 ± 0.55	39.56 ± 0.58	33.6 ± 0.40
C-23 (30 mg/kg)	9.5 ± 0.40^***^	8.36 ± 0.50^***^	9.4 ± 0.25^***^	15.63 ± 0.47^***^	5.8 ± 0.60^***^
C-31 (30 mg/kg)	21.6 ± 0.70^***^	31.73 ± 0.66^***^	21.4 ± 0.60^***^	30.16 ± 0.50^**^	26.56 ± 0.58^**^
C-44 (30 mg/kg)	30.4 ± 0.60^***^	38.56 ± 0.45^***^	30.33 ± 0.60^***^	36.56 ± 0.51^*^	32.6 ± 0.45^ns^

The results representing the inhibition at each time point, six mice per group (*n* = 6). Statistical analysis was performed using ANOVA followed by Bonferroni’s post hoc test. Significance levels (using Graph Pad Prism 5) for histamine-induced inflammation are denoted as ***P < 0.001, **P < 0.01, *P < 0.05, and *ns* for non-significant compared with chlorpheniramine maleate (CPM)

The proposed mechanism for these compounds might involve the inhibition of histamine H1 receptor modulation; however, this interpretation is based on functional outcomes rather than direct receptor-binding or signaling assays. By blocking these receptors, the compounds reduce the release of histamine and other inflammatory mediators, thereby mitigating the inflammatory response (Veber et al. 2002). This mechanism underscores the potential of compounds C-23, C-31, and C-44 as novel anti-inflammatory agents.

#### Bradykinin-induced inflammation

Bradykinin, a physiologically active nine-amino-acid oligopeptide, plays a critical role in inflammation, mainly through its receptor, prominently expressed during tissue damage (Paterson et al. 2013) and (Owemidu et al. 2018). Our current study evaluated the anti-inflammatory efficacy of compounds C-23, C-31, and C-44 following bradykinin treatment. The mice for the bradykinin-induced oedematogenic activity were divided into four (4) different groups. Each group has 6 mice (*n* = 6) in it. DMSO at a concentration of 10% was given to Group I. Bradykinin at a concentration of 20 µg/ml was administered to Group II. Bradykinin with icatibant (1 mg/kg) was given to Group III. Bradykinin with pure synthetic chemical C-23, C-31, and C-44 (30 mg/kg) was received by Group IV. The results indicated that these compounds exhibited moderate activity, with inhibition levels of 7.63 ± 0.47%, 13.16 ± 0.50%, and 17.7 ± 0.43% at the first-hour post-administration (Table 6). This suggests that while these compounds have some anti-inflammatory potential, their effects are not robust. In contrast, the bradykinin receptor antagonist icatibant (HOE 140) showed potentinhibition of 66.13 ± 0.35% after 1 h of administration and maintained efficacy from 1 to 5 h. The superior performance of icatibant highlights the importance of targeting bradykinin receptors for effective anti-inflammatory action.

**Table 6:** Potential of compounds when inflammation was induced by bradykinin

Treatment/dose (mg/kg)	% Inhibition of paw oedema				
	1st h	2nd h	3rd h	4th h	5th h
Negative control	2.45 ± 0.27	2.13 ± 0.13	1.90 ± 0.35	1.71 ± 0.01	2.09 ± 0.41
HOE 140 (1 mg/kg)	66.13 ± 0.35	61.7 ± 0.45	64.6 ± 0.48	53.22 ± 0.52	48.66 ± 0.46
C-23 (30 mg/kg)	7.63 ± 0.47^***^	6.16 ± 0.40^***^	5.4 ± 0.52^***^	4.53 ± 0.44^***^	2.84 ± 0.31^***^
C-32 (30 mg/kg)	13.16 ± 0.50^***^	15.56 ± 0.62^***^	12.5 ± 0.45^***^	5.63 ± 0.47^***^	4.64 ± 0.46^***^
C-44 (30 mg/kg)	17.7 ± 0.43^***^	18.26 ± 0.45^***^	16.63 ± 0.47^***^	10.53 ± 0.45^***^	11.94 ± 0.52^***^

The results represent inhibition at each time point, six mice per group (*n* = 6). Data were analyzed using ANOVA followed by Bonferroni’s post hoc test to determine statistical significance, with results symbolized as ***P < 0.001 compares with HOE 140 (using Graph Pad Prism 5)

The proposed mechanism for the moderate anti-inflammatory effects of C-23, C-31, and C-44 might involve partial inhibition of the bradykinin B_2_ receptor, which is responsible for mediating bradykinin-induced inflammation (Table 6). The partial efficacy observed suggests limited interaction with the bradykinin B2 receptor, inferred from pharmacological response patterns reducing the release of pro-inflammatory cytokines and decreasing vascular permeability, reducing oedema and inflammation. However, their partial efficacy compared to icatibant suggests that these compounds may have a weaker binding affinity or a different interaction profile with the bradykinin receptor.

#### Inflammation induced by prostaglandin E2

Arachidonic acid (A.A.) is a vital component of all cell membrane phospholipids, playing a crucial role in the inflammatory response (Rossi et al. 2010). Cyclooxygenase (COX) is an enzyme involved in the metabolism of arachidonic acid, leading to the production of potent pro-inflammatory mediators, such as prostaglandins (Funk 2001).

In our current investigation, 0.9% normal saline solution was given to Group I as a negative control. Prostaglandin 0.01 µg/ml was administered to Group II (PGE_2_). Prostaglandin and celecoxib (50 mg/kg) were administered to Group III. Compound C-44 demonstrated the highest efficacy in inhibiting prostaglandin E_2_, achieving a 58.63 ± 0.44% inhibition (*P < 0.05) 1 h post-prostaglandin administrationand 80.1 ± 0.56 after 4th h. This effect is consistent with a possible interaction with the COX–prostaglandin pathway, as inferred from in vivo inhibition profiles and comparison with celecoxib. This potentinhibition suggests that C-44 effectively modulates the inflammatory pathway by targeting COX activity, thereby reducing the synthesis of prostaglandin E_2_, a key mediator in inflammation. The ability of C-44 to substantially decrease prostaglandin levels highlights its potential as a potent anti-inflammatory agent (Table 7). Further mechanistic studies are warranted to elucidate its precise mode of action and optimize its therapeutic application.

**Table 7:** Potential compounds when inflammation was induced by prostaglandin E_2_

Treatment/dose mg/kg	% Inhibition of paw oedema				
	1st h	2nd h	3rd h	4th h	5th h
Negative control	2.30 ± 0.17	2.37 ± 0.15	2.53 ± 0.30	2.60 ± 0.41	2.75 ± 0.11
Celecoxib (50 mg/kg)	64.66 ± 0.46^***^	75.63 ± 0.62^***^	82.26 ± 0.62^***^	88.36 ± 0.46^***^	79.23 ± 0.55^***^
C-23 (30 mg/kg)	45.63 ± 0.62^***^	49.52 ± 0.48^***^	51.66 ± 0.61^***^	43.13 ± 0.50^***^	36.63 ± 0.44^***^
C-31 (30 mg/kg)	51.56 ± 0.53^***^	57.76 ± 0.30^***^	69.64 ± 0.42^***^	72.5 ± 0.45^***^	61.13 ± 0.31_***_
C-44 (30 mg/kg)	58.63 ± 0.44^**^	66.56 ± 0.40^**^	73.56 ± 0.56^**^	80.1 ± 0.56^**^	68.42 ± 0.40^***^

The percentage inhibition exhibited by the tested compounds (30 mg/kg) and celecoxib (50 mg/kg) in prostaglandin E2 (PGE2)-induced inflammation is detailed below. The results representing inhibition at each time point, six mice per group (*n* = 6). Data were analyzed using ANOVA followed by Bonferroni’s post hoc test to determine statistical significance, with results indicated as ***P < 0.001, **P < 0.01 comparesd with celecoxib (using Graph Pad Prism 5)

#### Involvement of leukotriene

Leukotrienes, potent inflammatory mediators derived from arachidonic acid through the action of the 5-lipoxygenase enzyme, play a crucial role in the inflammatory response (Wang et al. 2018). These mediators are produced by various inflammatory cells, including basophils, neutrophils, mast cells, eosinophils, and monocytes (Longley et al. 1995). In this study, normal saline 0.9% was given to Group I as a negative control. The phlogistic agent leukotriene (10 µg/ml) was given to Group II. Leukotriene and a positive control drug montelukast (20 mg/kg B. wt) were given to Group III. The results indicated that the compound C-44 exhibited potent, dose-dependent anti-inflammatory activity, reducing 53.6 ± 0.513% in leukotriene-induced inflammation at the first hour post-injection and 62.26 ± 0.55. These findings suggest that C-44 may exert potential modulation of leukotriene-mediated pathways, thereby inhibiting the inflammatory response. The specific mechanisms underlying the anti-inflammatory effects of C-44 could involve the inhibition of leukotriene synthesis or receptor antagonism. By targeting the 5-lipoxygenase pathway or leukotriene receptors, C-44 may effectively reduce the recruitment and activation of inflammatory cells, thus mitigating the overall inflammatory process. This study underscores the potential of C-44 as a therapeutic agent in treating inflammatory conditions, highlighting its potent and dose-responsive capabilities in modulating leukotriene-mediated inflammation (Table 8).

**Table 8:** Potential of compounds in leukotriene-induced inflammation

Treatment/dose (mg/kg)	% Inhibition of paw oedema				
	1st h	2nd h	3rd h	4th h	5th h
Negative control	2.74 ± 0.02	2.54 ± 0.03	2.31 ± 0.03	2.00 ± 0.03	2.07 ± 0.02
Montelukast (10 mg/kg)	59.5 ± 0.55^***^	66.72 ± 0.38^***^	70.10 ± 0.40^***^	63.53 ± 0.45^***^	47.66 ± 0.41^***^
C-23 (30 mg/kg)	35.7 ± 0.36^***^	31.32 ± 0.62^***^	40.33 ± 0.35^***^	38.23 ± 0.58^***^	18.62 ± 0.52^***^
C-31 (30 mg/kg)	48.46 ± 0.65^***^	49.56 ± 0.45^***^	54.26 ± 0.37^***^	49.43 ± 0.60^***^	30.66 ± 0.49^***^
C-44 (30 mg/kg)	53.6 ± 0.51^***^	56.56 ± 0.51^***^	62.26 ± 0.55^***^	57.53 ± 0.45^***^	39.44 ± 0.68^***^

The results demonstrate the degree of inhibition at each data point. Each experimental group consisted of six mice (*n* = 6). Data analysis was conducted using ANOVA and the Bonferroni post hoc test for multiple comparisons. Statistical significance (using Graph Pad Prism 5) was denoted as ***P < 0.001 compared with montelukast

#### Pharmacokinetic study

During our study, pharmacokinetic properties are also evaluated, like drug-like properties, physicochemical properties, water solubility and lipophilicity, bioavailability, and CNS effect.

#### Drug-like properties

The drug-like quality of a compound is significantly influenced by its drug-delivery mechanism. According to Lipinski’s Rule of Five (RO5), four fundamental physical and chemical properties are critical for substances intended for oral administration: a log *P* value ≤ 5, no more than five hydrogen bond donors, a molecular weight ≤ 500 Da, and no more than ten hydrogen bond acceptors (Lipinski 2004) and (Ahmad et al. 2023). Oral medications typically benefit from fewer hydrogen bond donors and acceptors and a limited number of rotatable bonds.

All synthesized derivatives in this study conform to Lipinski’s Rule of Five, indicating their potential suitability for oral drug development. The structures lack aromatic rings, and heavy atoms range from 14 to 16 (Table 9).

**Table 9:** Calculation of physicochemical properties of newly synthesized derivatives of succinimide using the Swiss ADME database

Ligands	Molecular formula	M.W. (g/mol)	TPSA (A^2^)	M.R	nHBD	nHBA	nRB	F. Csp^3^	nAHA	nHA
C-23	C_9_H_10_N_2_O_3_	194.19	87.03	50.33	1	4	3	0.56	0	14
C-31	C_9_H_10_N_2_O_4_	210.19	87.47	51.51	0	5	3	0.56	0	15
C-44	C_10_H_12_N_2_O_4_	224.21	87.47	56.31	0	5	4	0.60	0	16

*TPSA* represents topological polar surface area, *MR* molar refractivity, *nHBD* no. of H-bond donors, *nHBA* no. of H-bond acceptors, *nRB* no. of rotatable bonds, *Csp*^*3*^ fraction of sp^3^ hybridized carbons, *nAHA* no. of aromatic heavy atoms, *nHA* no. of heavy atoms, *M.W* molecular weight

#### Physicochemical properties

Physicochemical properties of the selected chemical compounds were evaluated. C-44 has a molecular weight of 224.21, C-31 has a molecular weight of 210.19, while C-23 has a molecular weight of 194.19 (Table 9).

#### Water solubility and lipophilicity

The lipophilicity and solubility of these compounds are crucial factors in determining their drug-like properties. The log Po/w values are proportional to the log S values, indicating relative solubility in water. Log S, representing water solubility, should ideally be within the − 4.05 log mol/L range. Water solubility and lipophlicity, solubility class and Log S values of our novel compounds were determined by the Swiss ADME database (Table 10).

**Table 10:** Calculation of water solubility and lipophilicity of newly synthesized derivatives of succinimide using the Swiss ADME database

Ligands	Water solubility						Lipophilicity
	Log S (SILICOS-IT)	Solubility class	Log S (all)	Solubility class	Log S (ESOL)	Solubility class	Consensus log P_o/w_
**C-23**	− 1.47	Soluble	− 0.46	Very soluble	− 0.29	Very soluble	− 0.13
**C-31**	− 0.48	Soluble	− 0.34	Very soluble	− 0.31	Very soluble	− 0.28
**C-44**	− 0.88	Soluble	− 0.72	Very soluble	− 0.56	Very soluble	− 0.03

#### Pharmacokinetic evaluation

The bioavailability score for all derivatives remained consistent at 0.55, a critical metric to be determined before advanced investigation (Table 11). A bioavailability score greater than 10% in mice is promising (Martin 2005).

**Table 11:** Pharmacokinetics evaluation of succinimide derivatives using the Swiss ADME database

Ligands	CYP3A4 inhibitors	CYP2D6 inhibitors	CYP2C9 inhibitors	CYP2C19 inhibitors	CYP1A2 inhibitors	P-gp substrate	BBB permeant	Bio-availability Score	Gestro intestinal absorption
C-23	No	No	No	No	No	No	No	0.55	High
C-31	No	No	No	No	No	No	No	0.55	High
C-44	No	No	Yes	No	No	No	No	0.55	High

The SwissADME database assigns synthetic accessibility scores ranging from 2.53 to 2.81, indicating straightforward synthesis processes. A high synthetic accessibility score suggests that the synthetic procedure is more complex (Ahmad et al. 2023). The possible drug interaction is with ibuprofen, warfarin, phenytoin, and losartan (Schwarz 2003).

#### Bioavailability and CNS effects

**Fig. 2:**
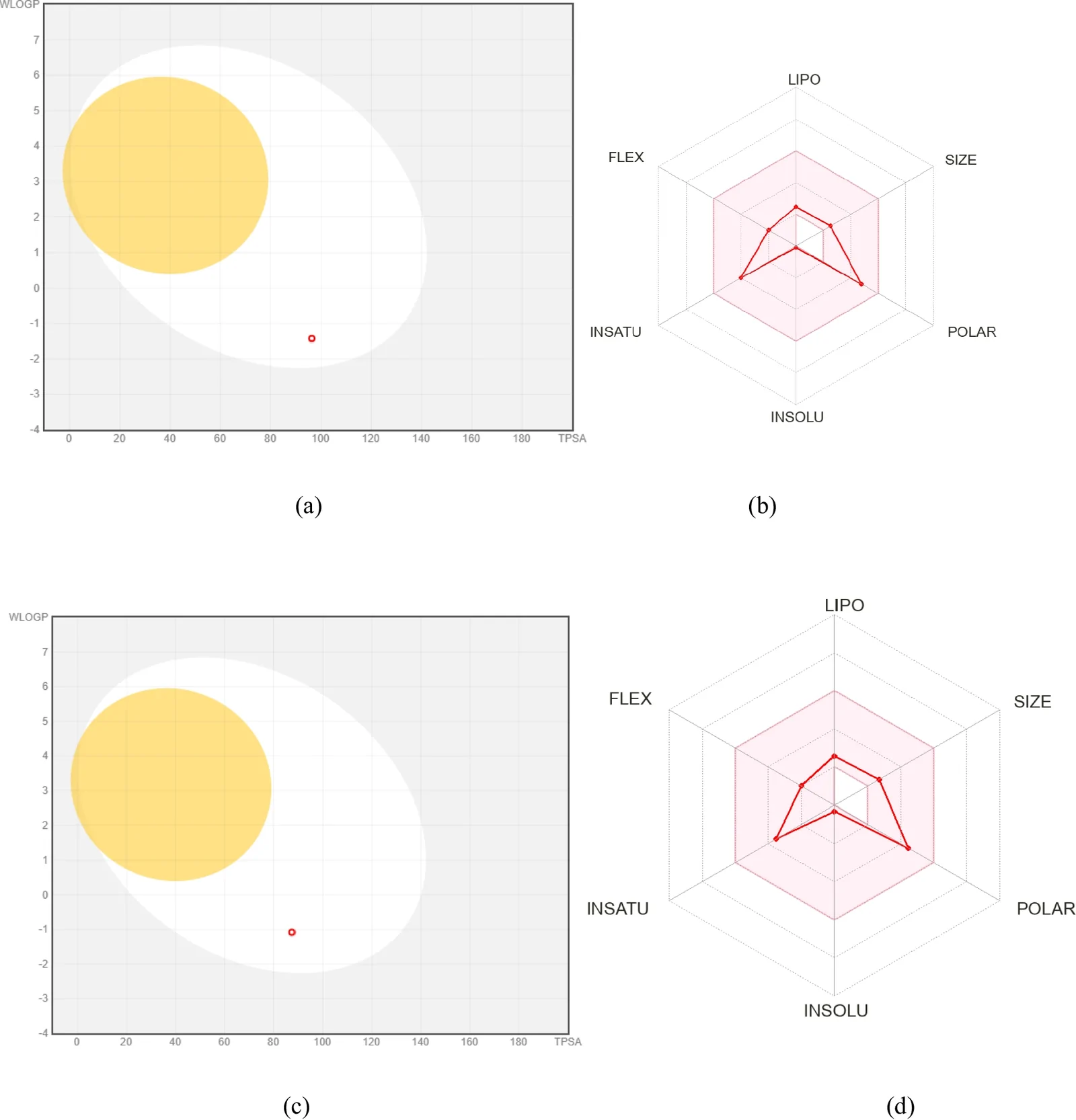

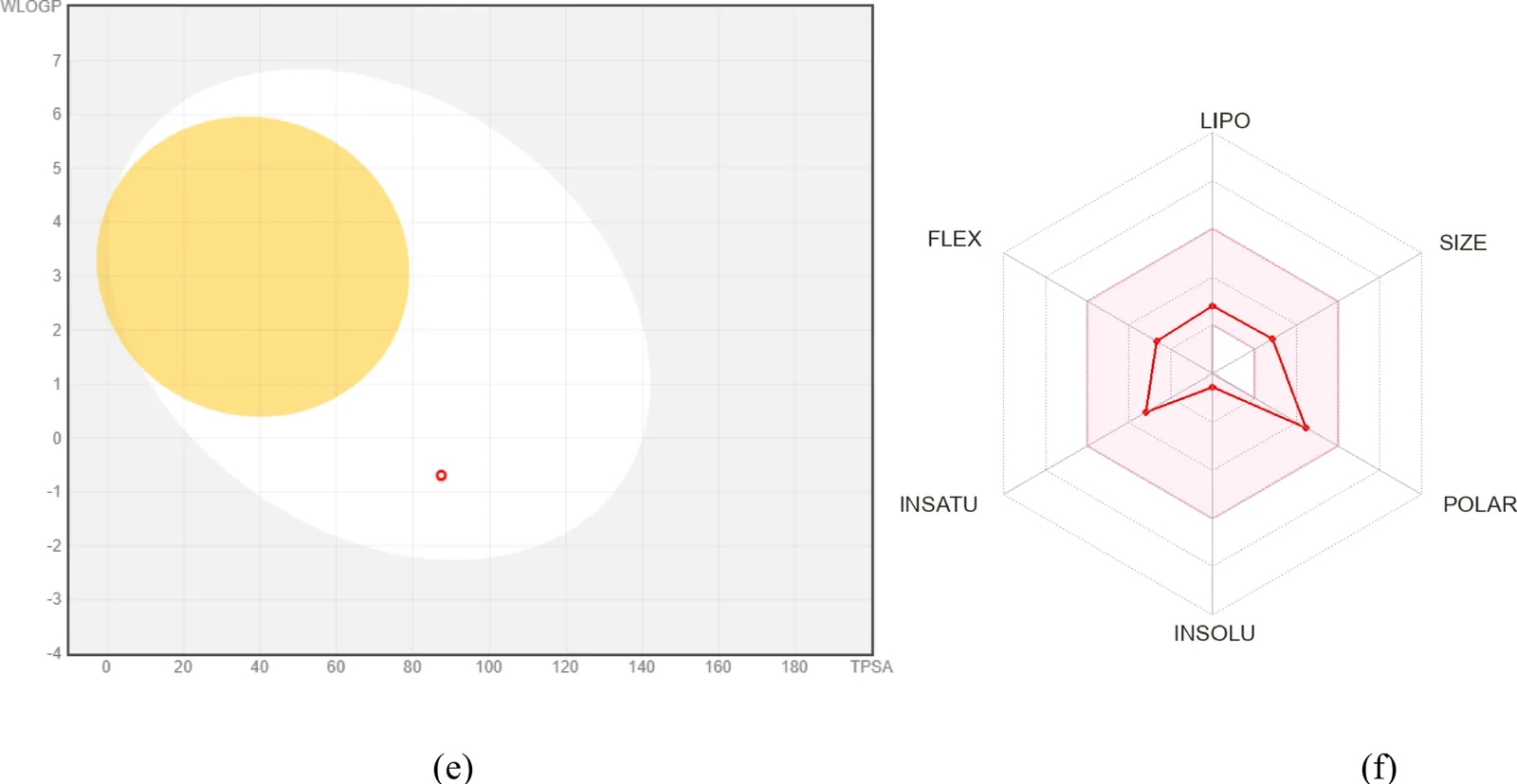
Bioavailability radar and BOILED-Egg plots of compounds C-23, C-31, and C-44. Panels (**b**), (**d**), and (**f**) show radar charts illustrating the physicochemical space (pink zone) and predicted oral bioavailability (red line). Panels (**a**), (**c**), and (**e**) present BOILED-Egg plots generated by the SwissADME tool

Bioavailability radar charts for C-23, C-31, and C-44 were given in Fig. 2. The pink zone represents the physicochemical space for oral bioavailability, and the red line represents the oral bioavailability properties.

These compounds’ high intestinal absorption (HIA) and low blood–brain barrier (BBB) permeability indicate minimal CNS effects. The BOILED-Egg plots illustrate the compounds’ BBB penetration and GI absorption, where the white zone represents HIA and the yellow zone represents BBB penetration. None of the compounds is a P-gp substrate, indicating they are not subject to the P-gp efflux process, often exploited by cancer cell lines to establish drug resistance (Fig. 2).

#### Medicinal chemistry and drug-likeness

All synthesized derivatives meet the criteria for drug-likeness, with minimal violations, demonstrating their potential as viable oral medications (Table 12).

**Table 12:** Medical chemistry and drug likeness of C-44, C-31, and C-23

Ligands	Medicinal chemistry					Drug likeness rules			
	Muegge	Egan	Veber	Ghose	Lipinski	Synthetic accessibility	Lead likeness	Brenk	PAINS
**C-23**	No	Yes	Yes	No; 1 violation WLOGP < − 0.4	Yes	2.53	No.; 1 violation: MW < 250	1 alert: phthalimide	zero alert
**C-31**	Yes	Yes	Yes	No; 1 violation: WLOGP < − 0.4	Yes	2.71	No.; 1 violation: Mw < 250	1 alert: phthalimide	Zero alert
**C-44**	Yes	Yes	Yes	No; 1 violation: WLOGP < − 0.4	Yes	2.81	No.; 1 violation: Mw < 250	1 alert: phthalimide	Zero alert

*PAINS* pan assay interference compounds

The relationship between predicted pharmacokinetic behavior and in vivo efficacy; a comparative summary of oral bioavailability scores and anti-inflammatory potency was prepared (Table 13). All tested compounds exhibited an identical SwissADME-predicted bioavailability score (0.55); however, marked differences in maximal oedema inhibition were observed. Among the tested compounds, C-44 showed the highest in vivo anti-inflammatory activity, followed by C-31 and C-23, suggesting that differences in pharmacological efficacy are primarily driven by pharmacodynamic rather than predicted oral bioavailability parameters.

**Table 13:** Comparison of predicted bioavailability and in vivo anti-inflammatory potency

Compound	Bioavailability score (SwissADME)	Dose range tested (mg/kg)	Max. % inhibition*	Estimated potency
C-23	0.55	10–30	~ 40%	Moderate
C-31	0.55	10–30	~ 49%	Good
C-44	0.55	10–30	~ 56%	Highest

## Discussion

This study aimed to evaluate the in vivo anti-inflammatory potential of three newly synthesized cyanoacetate derivatives of succinimide using two assays: carrageenan-induced paw oedema and arachidonic acid-induced ear oedema. After assessing their basic anti-inflammatory activity, the most potent compounds were subjected to mechanistic studies by inducing inflammation through different mediators, including histamine, bradykinin, prostaglandin, and leukotriene. Given the limitations and adverse effects associated with existing anti-inflammatory therapies, these investigations aimed to identify multitarget candidates with favourable efficacy, safety, and drug-like properties.

In our research among the tested compounds, C-44 demonstrated markedanti-inflammatory activity in the carrageenan-induced paw oedema assay at 10, 20, and 30 mg/kg doses. At 30 mg/kg, C-44 showed inhibition values of 46.4 ± 0.81, 54.2 ± 0.72, 52.67 ± 0.86, 55.6 ± 0.60, and 56.5 ± 0.55, closely approaching the efficacy of the standard drug diclofenac sodium (10 mg/kg), which exhibited a maximum inhibition of 57.3 ± 1.25 (Table 3). Similarly, in the arachidonic acid-induced ear oedema assay, C-44 at 30 mg/kg achieved a maximal inhibition value of 64.66 ± 0.58, comparable to the standard drug nimesulide (Table 4). Although C-44 showed the highest activity, C-23 and C-31 also demonstrated consistent, dose-dependent effects, supporting the relevance of the succinimide scaffold as a whole.

The mechanistic studies revealed that C-44 exhibited mild inhibition in the histamine-induced inflammation assay, with an inhibition value of 38.56 ± 0.45 at the 2nd hour, whereas the standard anti-histaminic drug chlorpheniramine maleate showed markedinhibition of 72.36 ± 0.56 at the 1 st hour (Table 5). This indicates that C-44 does not predominantly follow the histaminic pathway. For the bradykinin-induced inflammation assay, compounds C-23, C-31, and C-44 showed poor activity with inhibition values of 7.63 ± 0.47, 13.16 ± 0.50, and 17.7 ± 0.43 respectively, at the 1 st hour, compared to the standard drug icatibant (HOE 140), which showed an inhibition of 66 ± 0.35 at first hour. This suggests that these compounds do not considerably interact with the bradykinin pathway. In the prostaglandin E2-induced oedema assay, C-44 exhibited excellent inhibition at the 4th hour with a value of 80.1 ± 0.56% (*P* < 0.01), close to the standard drug celecoxib, which showed an inhibition of 88.36 ± 0.46 at 4th hour (Table 6). Compounds C-23 and C-31 also demonstrated markedactivity, indicating that these compounds follow the prostaglandin E_2_ pathway. This observation supports a COX-related mechanism consistent with the pharmacological profile of succinimide derivatives reported in earlier studies (Pervaiz et al. 2022). In the leukotriene-induced anti-inflammatory assay, C-44 showed an SEM value of 62.26 ± 0.550 at the 3rd hour, compared to the standard montelukast, which had an SEM value of 70.1 ± 0.40. C-31 and C-23 also exhibited notable activity, suggesting that these compounds follow the leukotriene pathway (Table 8). Collectively, these results indicate a multitarget anti-inflammatory profile involving both prostaglandin and leukotriene signaling. The C-44 and other derivatives showed notable inhibition in prostaglandin and leukotriene-induced assays, and inhibition in histamine and bradykinin-induced assays was relatively weak. Therefore, the exact contribution of each pathway cannot be quantitatively determined from the current data. Molecular docking studies, pathway-specific enzyme assays, or gene/protein expression analyses were not performed, which limits precise mechanistic conclusions. Future studies are required to confirm the relative contributions of prostaglandin, leukotriene, histamine, and bradykinin pathways. Overall, these results suggest a multitarget anti-inflammatory profile, with predominant effects on eicosanoid-mediated pathways, while activity through other mediators remains minor.

The “drug-like” quality of compounds is critically determined by their physicochemical properties and drug delivery mechanism. Lipinski’s Rule of Five (RO5) outlines four key characteristics for oral drugs: hydrogen bond acceptors (≤ 10), hydrogen bond donors (≤ 5), log *P* (≤ 5), and molecular weight (≤ 500) (Lipinski 2004). These criteria suggest that fewer hydrogen bond acceptors, donors, and rotatable bonds are preferred for oral medicines (Ahmad et al. 2023). All synthesized derivatives in this study meet Lipinski’s criteria, making them suitable candidates for oral medication development. These derivatives lack aromatic rings, with heavy atoms ranging between 14 and 16 (Table 9).

The proportion of sp3 hybridized carbons (Fsp3) in the succinimide derivatives ranges from 0.56 to 0.60, indicating a favourable degree of carbon saturation and molecular complexity (Table 9). The optimal Fsp3 range is 0.42, as it is found in over 84% of marketed drugs (Kombo et al. 2013). These derivatives have 3 to 4 rotatable bonds, aligning with the recommendation that rotatable bonds be fewer than 10 to ensure good oral bioavailability (Veber et al. 2002). The molar refractivity, another critical parameter, falls between 50.33 and 56.31. Additionally, a TPSA (topological polar surface area) of > 140 Å^2^ is associated with poor absorption (< 10% fractional absorption), but the succinimide derivatives have TPSA values within the favourable range of 87.03 to 87.47 (Table 9), among which C-44 posses heighest TPSA value of 87.47 and also exhibited the strongest inhibition across time points**,** particularly at 30 mg/kg during in vivo anti-inflammatory assays (Table 3).

The Swiss ADME database calculates the octanol/water partition coefficient (Log Po/w), which reflects lipophilicity based on polarity, molecular size, and hydrogen bonding. Log Po/w values range from − 0.28 to − 0.03 for the succinimide derivatives (Table 10), suggesting good water partitioning. The ideal Log P range for optimal lipophilicity is 0 < Log *P* < 3 (Bitew et al. 2021). Although slightly below the conventional range, these negative Log *P* values indicate enhanced aqueous solubility, which may favor oral absorption and formulation development. Pharmacokinetics (PK) are crucial in drug development, as they influence the pharmacological activity of a drug. The Swiss ADME database revealed that all derivatives have substantial gastrointestinal absorption (Daina et al. 2017). None of the compounds is expected to cross the blood–brain barrier, reducing the likelihood of central nervous system toxicity. None of the derivatives is substrates for P-glycoprotein, minimizing potential drug-drug interactions (Qiu et al. 2021). C-44 is identified as a CYP2C9 inhibitor but does not inhibit other CYP P450 isoforms, such as CYP2D6, CYP3A4, CYP1A2, and CYP2C19 (Table 11). This selective metabolic interaction profile supports a reduced risk of broad cytochrome-mediated adverse interactions.

All derivatives have similar bioavailability scores of 0.55, consistent with acceptable drug-likeness properties (Martin 2005). These scores were determined before any additional testing. None of the compounds violates the drug-likeness rules Lipinski, Veber, and Egan set. However, C-23 violates the Muegge rule, while C-31 and C-44 comply. All three derivatives violate a single Ghose rule because their WLOG *P* values are less than − 0.4. Despite this, their consensus Log Po/w and Log S values indicate good water solubility, which is advantageous in drug development. Additionally, the succinimide derivatives did not trigger any PAINS (Pan-assay interference compounds) alerts (Table 12), indicating they are free from reactive compounds, α-screen artifacts, and frequent hitters, which could interfere with typical physiological functions (Adasme et al. 2021).

Structural alerts from the Swiss ADME database, based on chemical toxicity, reactivity, metabolic stability, or poor pharmacokinetics, identified a reactive phthalimide group in the succinimide compounds. However, this reactivity can be seen in current market medications and drugs in preclinical phases (Brenk et al. 2008). Although SwissADME classifies these compounds as having molecular weights slightly above typical lead-like space, this does not preclude their optimization through SAR-guided structural modification. Lead-likeness evaluations highlight a molecule’s potential as a lead compound in drug discovery and show that all three derivatives have molecular weights greater than 250. This is generally acceptable as these compounds were specifically designed for pharmacological action, and their functional groups can be modified to enhance pharmacological outcomes according to structure–activity relationships (SAR).

The derivatives were synthesized and characterized using the Swiss ADME database analysis. Synthetic accessibility scores ranged from 2.53 to 2.81, indicating that these compounds can be synthesized through simple, single-step reactions. A score of 10 would indicate a complex synthesis process (Ahmad et al. 2023).

## Conclusion

The present study demonstrated that the cyanoacetate derivatives of succinimide exhibit marked anti-inflammatory activity, as confirmed by the carrageenan-induced paw oedema and arachidonic acid-induced ear oedema in vivo assays. Comparative evaluation revealed a clear efficacy trend (C-44 > C-31 ≈ C-23), with C-44 showing the most pronounced anti-inflammatory response. Mechanistic studies suggested that these derivatives primarily exert their anti-inflammatory effects through the prostaglandin and leukotriene pathways, while exhibiting weak involvement in the bradykinin pathway. Conversely, these compounds exhibit minimal activity via the histamine pathway. Importantly, all tested compounds showed satisfactory safety and acute toxicity profiles.

## Supplementary Information

Below is the link to the electronic supplementary material.

ESM 1 (DOCX 278 KB)

## Data Availability

All source data for this work (or generated in this study) are available upon reasonable request.
