# Supplementary material for: Pharmacokinetic evaluation and in vivo anti-inflammatory potential of cyanoacetate derivatives of succinimide
Source: Naunyn Schmiedebergs Arch Pharmacol. 2026 Jul 22;399(13):21411–26. doi: 10.1007/s00210-026-05720-z (PMC13695591; doi:10.1007/s00210-026-05720-z)
Supplement: Supplementary file 1 [file 210_2026_5720_MOESM1_ESM.docx]

**NMRs of all compounds**

^1^HNMR Spectrum of Compound C-23

^13^CNMR Spectrum of Compound C-23

^1^HNMR Spectrum of Compound C-31

^13^CNMR Spectrum of Compound **C-31**

**^1^HNMR Spectrum of Compound** C-44

**^13^CNMR Spectrum of Compound** C-44
